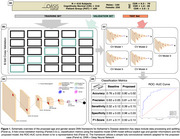# Gender and Age‐Aware Fully Convolutional Neural Network for Alzheimer's Detection

**DOI:** 10.1002/alz70856_101601

**Published:** 2025-12-25

**Authors:** Ninad Aithal IV, Neelam Sinha

**Affiliations:** ^1^ Indian Institue of Sciences, Bengaluru, Karnataka, India; ^2^ Centre for Brain Research (CBR), Indian Institute of Science, Bengaluru, Karnataka, India

## Abstract

**Background:**

Alzheimer's disease (AD) is a leading cause of mortality, significantly impacting global health. T1‐weighted MRI is an effective non‐invasive method for staging and identifying individuals at risk for AD. Demographic factors, particularly age and gender, significantly influence AD progression and detection. This study proposes and evaluates an age and gender‐informed deep learning model to differentiate cognitively normal (Clinical Dementia Rating [CDR] = 0.0) from cognitively impaired (CDR > 0.0) individuals using the OASIS‐1 dataset.

**Method:**

We utilized the OASIS‐1 dataset, comprising cross‐sectional data from 416 subjects aged 18 to 96, including individuals with early‐stage AD. Each scan underwent preprocessing steps: skull stripping, 12‐degree‐of‐freedom linear registration to a 2mm MNI152 template, and white stripe normalization. The images served as input to a six‐layer fully convolutional neural network. Our proposed model incorporates gender information as a one‐hot vector and age normalized to a 0‐1 range, concatenated with the feature vector from the T1w structural image at the penultimate layer. Using the PyTorch framework, we employed an 80‐20 train‐test split, followed by five‐fold cross‐validation on the training set, with each fold using 20% of the training data for validation and weighted binary cross‐entropy to address class imbalance. The model was trained with the SGD optimizer (learning rate: 1e‐4, batch size: 8) and early stopping (patience: 5) after 30 epochs.

**Results:**

The study included 316 cognitively normal and 100 cognitively impaired subjects. We analyzed the impact of incorporating explicit age and gender inputs by comparing the proposed model with a baseline model lacking these inputs. The proposed age and gender‐informed model achieved higher classification performance, with an 5 fold averaged accuracy of **80%**, specificity of **96%,** sensitivity of **39%** and ROC‐AUC of **0.88**, showing improvement over the baseline approach.

**Conclusion:**

This study underscores the importance of incorporating demographic information into data‐driven models for disease detection, as variables like age and gender play crucial roles in AD progression and susceptibility. Integrating such demographic factors improves model performance and enhances its potential for real‐world clinical applications.